# Bioactive Essential Oils from Cuban Plants: An Inspiration to Drug Development

**DOI:** 10.3390/plants10112515

**Published:** 2021-11-19

**Authors:** Lianet Monzote, Jesús García, Rosalia González, Marcus Tullius Scotti, William N. Setzer

**Affiliations:** 1 Department of Parasitology, Center of Research, Diagnostic and Reference, Institute of Tropical Medicine “Pedro Kouri”, Havana 11400, Cuba; 2 Research Network Natural Products against Neglected Diseases (ResNetNPND), University of Münster, 48149 Münster, Germany; mtscotti@ccae.ufpb.br; 3 Department of Pharmacy, Faculty of Natural and Exact Sciences, University of Oriente, Santiago de Cuba 90500, Cuba; jgadi1990@gmail.com; 4 Toxicology and Biomedicine Centre (TOXIMED), University of Medical Science, Santiago de Cuba 90400, Cuba; rosaliagonzalez.9110@gmail.com; 5 Post-Graduate Program in Natural and Synthetic Bioactive Products, Federal University of Paraíba, João Pessoa 58051-900, Brazil; 6 Department of Chemistry, University of Alabama in Huntsville, Huntsville, AL 35899, USA; 7 Aromatic Plant Research Center, 230 N 1200 E, Suite 100, Lehi, UT 84043, USA

**Keywords:** Cuba, essential oil, chemical composition, biological activity, antiprotozoal, antibacterial, antifungal, cytotoxic

## Abstract

Aromatic plants and essential oils are important agents as complementary and alternative medicines in many cultures and geographical locations. In this review, a literature search on essential oils from Cuba, their chemical compositions, and their pharmacological properties was carried out. Out of 171 published scientific articles on essential oils of Cuban plants, a total of 31 documents, focused on both chemical composition and pharmacological properties, were considered for this review. In general, an increase in articles published in the last decade was noted, particularly in recognized international journals in English. Myrtaceae and Piperaceae were the most representative families collected in the occidental area of the country. Leaves and aerial parts were predominantly used, while a wide and variable number of components were identified, including terpenes, aliphatic derivatives, sulfur-containing compounds, phenylpropanoids, alkaloids and amine-type compounds. Finally, different biological activities were reported such as antiprotozoal, antibacterial, antifungal, cytotoxic, anthelmintic, larvicidal and insecticidal. In conclusion, we encourage further studies that would promote the use of essential oils from Cuban plants in new pharmaceutical products.

## 1. Introduction

Natural products have served throughout history as a rich source of compounds that have found many applications to human life and continued to play a significant role in the drug discovery and development process [1]. During the last decade, an increase in the studies of natural products has been observed due to the interest of the scientific community towards complementary and alternative medicine, with the new hope of reducing the unwanted effects of modern medicine [2]. In this sense, the survey reported by Newman and Cragg [3] pointed to the fact that many drugs on the market are of natural origins. Among them, plants were found to still be the main source of new natural compounds [4].

Plants generally produce a diverse range of bioactive compounds, which have been widely used in clinical practice [5]. In particular, aromatic and medicinal plants have been the principal candidates for this task because of the long history of the successful use of folk medicine to treat different human diseases [6]. Aromatic plants naturally synthesize some secondary metabolites that can be isolated as essential oils (EOs), which are complex mixtures, mostly constituted of terpenoids that are biosynthesized by the mevalonate pathway, but may also be composed of phenylpropanoids [7].

Currently, more than 3000 essential oils (EOs) have been characterized, of which around 300 are commercialized and frequently used in cosmetics and flavors as well as in the food industries as spices or to prepare beverages [5,8,9]. During the late 20th century, EOs gained prominence, and have proven to be very popular in the 21st century [6]. The increased interest in the use of EOs has been established through the need to address their specific compositions and chemical structures, and by the extensive market for EOs, which has been driven by the growing demand for natural products that have little or no alterations, in addition to raising awareness of health and well-being among consumers [10], mainly due to their relaxant [11] and medicinal properties [12]. In this regard, EOs constitute an important source of biologically active compounds with antimicrobial, insecticidal, herbicidal, antioxidant and anti-inflammatory activities [2,8,13], which have been demonstrated in dozens of investigations and review articles [6,7].

On the other hand, Cuba (an island located in Caribbean region) presents a rich source of botanical and chemical diversity, as well as a long knowledge of medicinal plants [14]. In this sense, Fuentes (1980s) reported 1124 medicinal plants grouped in 615 genera belonging to 148 families that are used to treat diverse ailments. However, these represent only 18.8% of species of Cuban flora [15]. As an addition to the ever-increasing contributions on Cuban plant potentialities and EO properties, this review provides an overview of chemical compositions in relation to pharmacological studies of EOs from Cuban plants with the aim of highlighting potential therapeutic products. 

## 2. Results and Discussion

### 2.1. Study Selection

**Figure 1:**
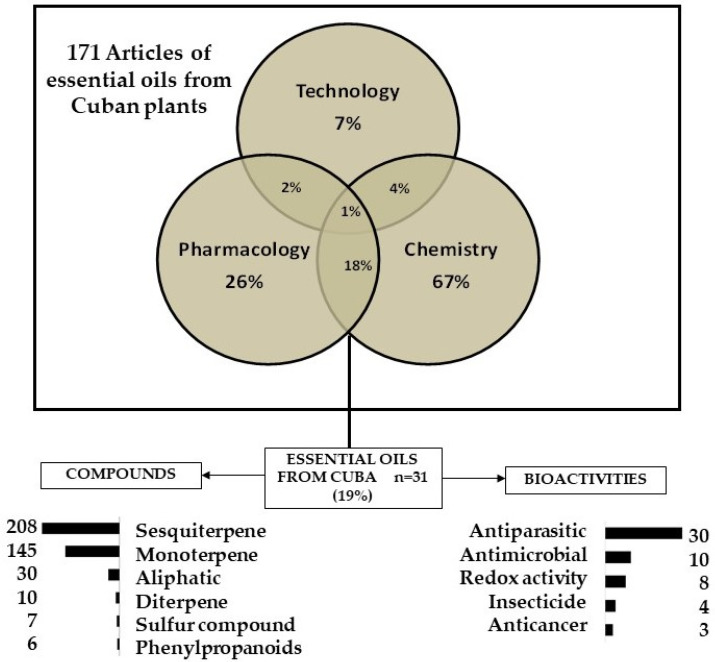
Scheme illustrating the main results retrieved of studies related to essential oils from Cuban plants.

A total of 349 published articles were obtained that mention the words “essential oil” and “Cuba” (Supplementary Figure S1). After the removal of duplicates, 171 articles pertaining to EOs from Cuban plants were retrieved. In general, a higher percentage (67%) of publications focused only in EO chemical characterization, followed by a minor percent (26%) of papers that described pharmacological properties without the identification of components (Figure 1). In this regard, out of the 310,000 described plant species worldwide, 15% have been phytochemically studied and only 6% pharmacologically investigated [16]. Our data corroborate that the chemical diversity should maximize the possibilities of finding useful compounds for human medical needs [4]. In the end, we selected 31 articles, which include both chemical composition analyses and pharmacological studies. Thus, the documents used in this review represent 19% of articles reporting on EOs from Cuba. As previously pointed out, this trend emphasizes the need for drug discovery from plants to involve multidisciplinary approaches, as claimed by several authors in previous reviews [16,17].

### 2.2. Bibliometric Analysis of Reports about Essential Oils from Cuban Plants

**Figure 2:**
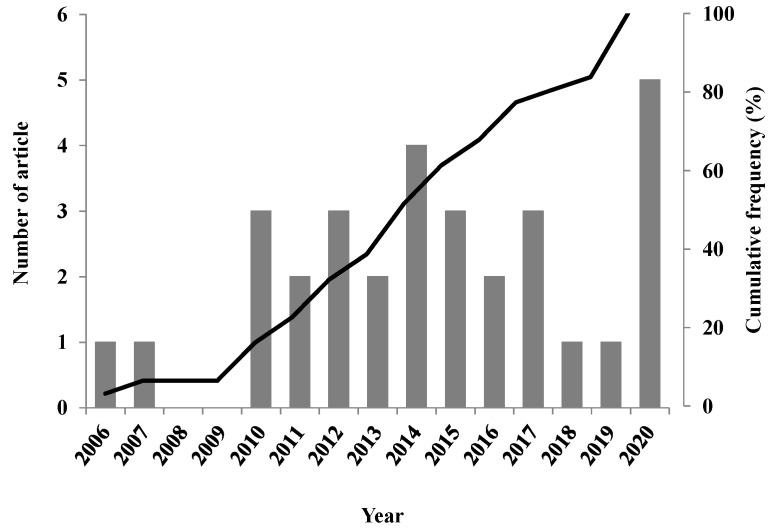
Evolution during the time of the included scientific articles (*n* = 31). **Bars:** Time course of publications showing absolute numbers; **Line:** Cumulative frequency.

The 31 selected articles in the covered area, published up until December of 2020, represented an increase in publication the last decade (Figure 2). Nevertheless, higher year-to-year variations were appreciated, with the most successful years being 2014 and 2020 with 4 and 5 published documents, respectively. Additionally, an increasing cumulative frequency could be a positive indicator of the research importance of the discovery of new therapeutic agents from Eos, although the most productive period was from 2010 to 2017 with a total of 22 articles (71%). 

The affiliations of the first author showed that only centers from 3 provinces were included in the 31 articles: Havana (71%), Las Villas (10%) and Santiago de Cuba (10%). The most productive centers were the Institute of Tropical Medicine Pedro Kourí (42%) and the Food Industry Research Institute (13%), both located in Havana. Nevertheless, important contributions were also made by Central University “Marta Abreu” of Las Villas (10%) and the University of Oriente (10%) of Villa Clara and Santiago de Cuba, respectively. Note that the first authors of three articles are foreign researchers, from Instituto Oswaldo Cruz, Brazil (6%), and the University of Alabama in Huntsville, USA (3%).

Although the official language of Cuba is Spanish, the vast majority of articles were published in English (97%) and only one document (3%) was published in Spanish. In this sense, the recognition of international journals that focus on the development of phytotherapeutics illustrates the increasing international reputation of this avenue of research (Table 1). In particular, *Natural Product Communications* (23%), *Journal of Essential Oil Research* (13%), *Chemistry and Biodiversity* (10%) and *Phytotherapy Research* (7%) were the most representative journals.

### 2.3. Chemical and Pharmacological Overview of Essential Oil from Cuban Plants

**Figure 3:**
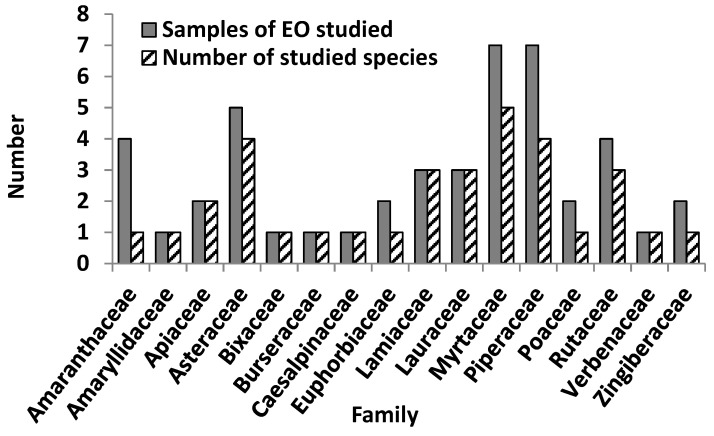
Samples and studies of essential oils from Cuban plants included in this study according to family.

As mentioned above, 31 articles that reported the chemical characterization of EOs from Cuban plants with biological activity were considered, with 33 plants and 45 samples. In general, two classic strategies were observed in the methods performed with the selected plants. In the first strategy, plants were selected based on pharmacognosy, ethnopharmacology or traditional knowledge criteria; while the second strategy was based on the use of modern pharmacology to screen and validate the use of plants as sources of drugs with extensive chemical diversity in secondary metabolites. In both cases, plant organs were collected in precise geo-localized sites, the botanical material was identified and authenticated in a recognized herbarium, and essential oil was extracted and chemically characterized, and subsequently, its biological activity was tested in different experimental biological models. In this compilation, 16 families and 33 species were included. The most representative families were Myrtaceae and Piperaceae with 7 reports, while 5 and 4 species or samples of EOs were studied, respectively (Figure 3). Asteraceae was also frequent, with 5 reports and 4 studied species. In some cases, more than one sample of the same plant was studied (Figure 3), as was the case of *Dysphania ambrosioides* (L.) Mosyakin & Clemants from the Amaranthaceae family (see Table 2).

Table 2 summarizes all plant species, with some characteristics that include collection site, organ of plant used to obtain the essential oil, and the main chemical compounds. Among them, the most studied plants were *D. ambrosioides*, with four and three samples, respectively. In addition, in correlation with the main institutions obtained from bibliometric results, most of the plants were collected in western Cuba (56%), mainly from Havana (38%) and Artemisa (19%) provinces. Other recognized research points were in Sancti Spiritus (19%) and Santiago de Cuba (9%), where two important natural reserves, Topes de Collantes and Siboney-Juticí Ecological Reserve, are located, respectively. 

In general, leaves (56%) and aerial parts (36%) are the most used plant organs (Table 2), in agreement with most of the essential oil studies conducted on leaves. Obviously, leaves are one of the most accessible parts of any plant, they are abundant, and they are renewable, which allows for preservation of the whole plant. This point is also valid for fruits or flowers, but their availability is subject to seasonality [49,50].

The extraction of EOs has been conducted through conventional hydrodistillation using a Clevenger apparatus, with a range of extraction times from 3 to 6 h. Subsequently, chemical compositions are generally analyzed by gas chromatography coupled with a mass spectrometric detector (GC-MS). The identification of the oil components is typically based on their retention indices (RI) determined by reference to a homologous series of *n*-alkanes, and by comparison of their mass spectral fragmentation patterns using MS library databases (e.g., the NIST database) and the RI values reported in the literature. 

**Figure 4:**
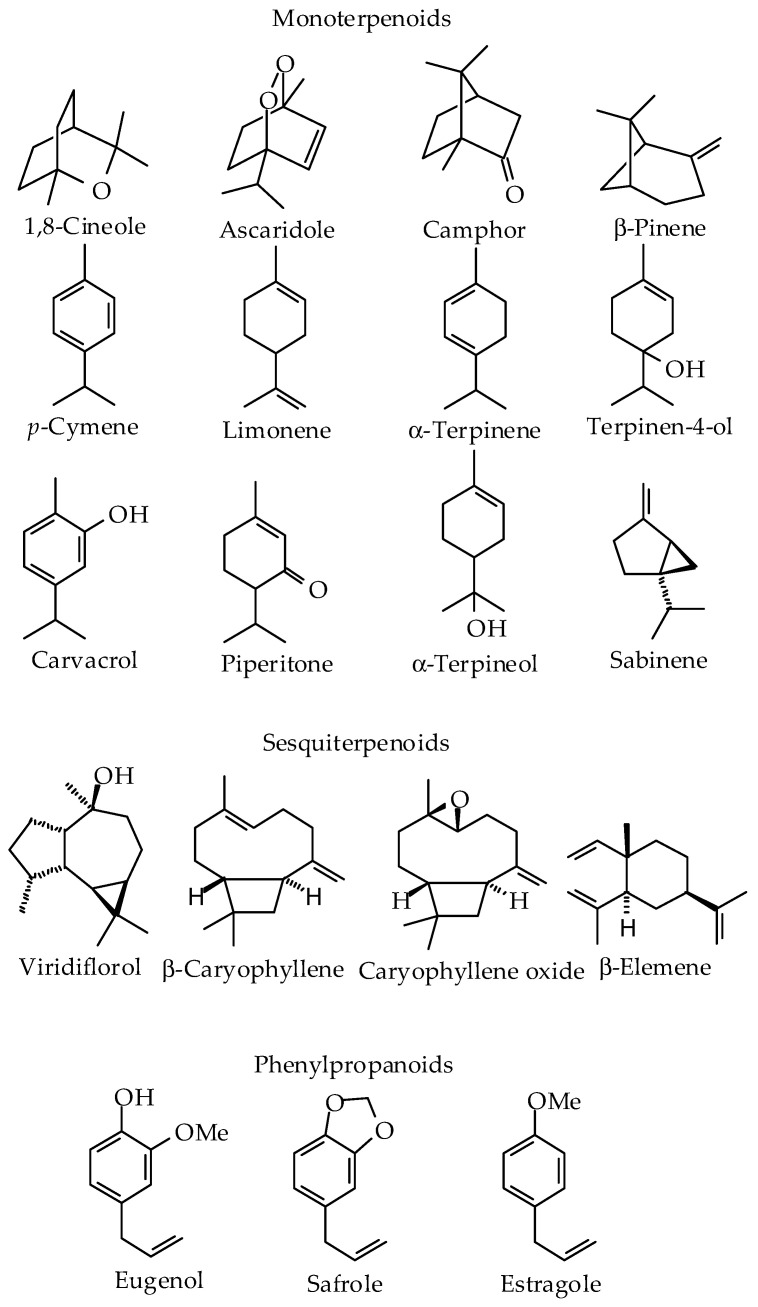
Chemical structures of the major components of essential oils from Cuba that were included in this study.

The chemical compositions of the analyzed EOs displayed a wide and variable number of compounds, with the main entities being known volatile compounds that are commonly found in essential oils. In Table 2, the main compounds are summarized (Figure 4). Among them, the most identified components were 1,8 cineole (also known as eucalyptol) and viridiflorol in six different essential oils. There were followed by ascaridole, camphor, *p*-cymene, eugenol, piperitone and terpinen-4-ol, present in four essential oils, while α-terpinene, limonene, safrole, α-terpineol and β-caryophyllene were reported in three oils. Finally, carvacrol, caryophyllene oxide, estragole, sabinene, β-elemene and β-pinene were identified only in two essential oils (see Figure 4). The remaining compounds were present in only one of the studied oils. 

The analysis of complete chemical composition (except traces) corroborates the high complexity and diversity of EOs. In this sense, 408 different compounds were identified with concentrations from 0.1%, which included 208 sesquiterpenes, 145 monoterpenes, 30 aliphatic derivatives, 10 diterpenes, 7 sulfur-containing compounds and 6 phenylpropanoids. An alkaloid and an amine-type compound were also observed. The compositions of EOs of this compilation also correspond with variable mixtures of terpenoid compounds, especially monoterpenes and sesquiterpenes, observed in other essential oils [7]. Taking into account the complete chemical compositions, the most common compounds identified were linalool (26 samples), *p*-cymene (24 samples), α-pinene and α-terpineol (22 samples), myrcene (20 samples), terpinen-4-ol and β-pinene (19 samples), γ-terpinene (18 samples), limonene (17 samples) and 1,8-cineole (16 samples). It is known that plants from different taxa can also synthesize identical although rather complex compounds. Gene duplication and neo-functionalization, leading to the extension of the existing metabolic pathways, are both part of the mechanisms that were identified in plants as responsible for the diversification of secondary metabolites together with the influence of ecological factors [4]. Nevertheless, 1,8-cineole, limonene, *p*-cymene, terpinen-4-ol, α-terpineol and β-pinene are among the most relevant compounds, as major components with high concentrations and as the more frequently identified components in EOs. However, some articles only mentioned the main compounds and did not summarize the complete chemical compositions of the EOs. Figure 4 represents the most abundant/relevant compounds among the studied EOs. 

Another interesting and explored point is the intraspecific chemical diversity that could occur in the plant due to the environment (localization, time of day, or season of the year) or the part of the plant from which the oil was obtained. In this sense, Moore et al. [51] pointed out not only the plant ontogeny, but also genetic and environmental variations as major sources of diversity for plant secondary metabolites. This intraspecific chemo-diversity, well described in plants producing volatile oils, has been explained by the existence of chemotypes. Factors such as moisture, salinity, temperature, or nutrition levels are known to influence the oil production, although the genotype could also significantly influence the chemotype [52]. In this sense, only one chemotype was observed for *Citrus sinensis* (L.) Osbeck [20,38], *Piper aduncum* L. [40,41], *Piper auritum* Kunth [41,43] and *Tagetes lucida* Cav. [24,25], while two chemotypes were observed for *D. ambrosioides* [18,19], *Croton linearis* Jacq. [28,29] and *M. leucadendra* [36,37]. Nevertheless, the chemical biodiversity should be analyzed in greater detail with a higher number of EOs extracted from Cuban plants. 

Over the past two decades, several bioactive EOs have been studied. From the 33 plant species studied, different biological activities were reported, which was possible due to EOs showing biological activities on different cell targets (Table 3). In particular, it is noteworthy to highlight the antiproliferative activity demonstrated by EOs, including, in order of importance: antiprotozoal (38%), antibacterial (20%), antifungal (11%), anticancer (5%), anthelmintic (3%), larvicidal (3%) and insecticidal (2%) activities. In terms of secondary metabolites, as was the case for EOs, the chemical diversity was linked with plant defense because these compounds are synthetized constitutively as part of normal plant development and are stored in specialized tissues [53].

The other additional biological characteristic studied was related to the redox effect, which displayed antioxidant activity (11%) and precursors of oxidative stress (2%). In addition, four essential oils (6%) did not show relevant biological activity.

The most notable activity of Cuban essential oils was the antileishmanial potential, due to twelve plants from different families, namely *Artemisia absinthium* L., *Bursera graveolens* (Kunth) Triana & Planch., *Bixa orellana* L., *C. linearis, D. ambrosioides, M. leucadendra, P. aduncum, P. auritum, Piper ossanum* Trel., *Plectranthus amboinicus* (Lour.) Spreng, *Pluchea carolinensis* (Jacq.) G. Don., *Phania matricarioides* (Spreng.) Griseb. and *T. lucida*, being active in vitro against *Leishmania* spp. The most promising was the essential oil from *D. ambrosioides* with an IC_50_ of 3.7 μg/mL and 4.6 μg/mL against promastigotes and amastigote of *Leishmania amazonensis*, respectively. In addition, the effectiveness of this oil was demonstrated against the cutaneous leishmaniasis in BALB/c mice [18]. 

In parallel, the antiplasmodial activity was also relevant in the studied series. In this case, essential oils from *P. aduncum* [40], *P. amboinicus* [32], *P. matricarioides* [22] and *P. ossanum* [54] were active against *Plasmodium falciparum*. Other reports based on the essential oil from *Alpinia zerumbet* (Pers.) B.L. Burtt and R.M. Smith [48] and *T. lucida* [25] also showed this pharmacological effect on *Plasmodium berghei*. 

Relevant antiprotozoal activity also includes the antitrypanosomal potential of essential oils from: (i) *C. linearis* against *Trypanosoma cruzi* [28]; (ii) *M. leucadendra* [36] and *P. amboinicus* [32] against *Trypanosoma brucei*; and (iii) *P. aduncum* [40], *P. matricarioides* [22], and *P. ossanum* [54] against both *Trypanosoma* species. Furthermore, the essential oils from *C. linearis* [28], *M. leucadendra* [36], *P. aduncum* [40], *P. amboinicus* [32], *P. matricarioides* [22] and *P. ossanum* [54] were able to inhibit the growth of more than one protozoal parasite. These results, demonstrating the broad antiprotozoal spectra of Cuban EOs, constitute a potential source for their use in endemic areas, where more than one infectious species is present. 

An increasing number of studies regarding the activity of EOs are also concerned with human parasites of major medical importance, including antileishmanial, antitrypanosomal and antiplasmodial potentialities [55,56,57]. Nevertheless, studies have been generally performed on in-vitro cultures of parasites, which necessitates further in vivo studies using animal models to validate the antiprotozoal potential. In this respect, we highlight the fact that all EOs tested in animal experimental models were carried out against cutaneous leishmaniasis in BALB/c mice. In this model, *A. absinthium, B. orellana, D. ambrosioides, M. leucadendra* and *P. carolinensis* were effective to control infection caused by *Leishmania amazonensis.* Leishmaniasis is a parasitic disease that is non-endemic to Cuba, although a few imported cases have been documented [58]. However, leishmaniasis is considered to be one of the most important tropical diseases by the World Health Organization (WHO) and presents high importance in the Americas due to an elevated number of leishmaniasis cases (accounting for two-thirds of the worldwide disease burden) and circulating *Leishmania* species [59]. 

In parallel, three of the previously mentioned essential oils with antiprotozoal activity, also showed in vitro activity on cancer cell lines. Nowadays, cancer is one of the deadliest diseases in the world, and was estimated to have caused 9.9 million deaths in 2020. Conventional treatments for cancer commonly involve mono-chemotherapy or a combination of radiotherapy and mono-chemotherapy. However, the negative side effects of these approaches have been extensively reported and have prompted the search for new therapeutic drugs. In this context, the scientific community started to look for innovative sources of anticancer compounds from natural sources [60]. In this sense, the oil from *B. graveolens* was active against the MCF-7 cancer cell line with an IC_50_ < 50 μg/mL [27] and *M. leucadendra* oil displayed inhibition of the malignant cell lines 22 Rv1, MCF-7 and EFO-21 and resistant sublines such as MCF-7/Rap and MCF-7/4OHTAMO with IC_50_ values ranging from 55 to 98 μg/mL [36]. Similar potentialities were observed with the oil from *P. amboinicus* against the human tumor-derived cell line MCF-7, showing IC_50_ < 50 μg/mL [32].

According to some authors, essential oils from Cuban plants have also demonstrated promissory antimicrobial activities. The antibacterial effects of *A. sativum* [20], *C. speciosus* [35], *L. camara* [47], *L. triandra* [33], *N. hihua* [34], *M. paniculata* [45], *O. vulgare* [20], *P. aduncum* [40], *S. aromaticum* [20] and *T. indica* [30], as well as the antifungal activities of *P. anisum* [20], *T. indica* [30] and *Z. pseudodumosum* [46], are examples that not only support the therapeutic potential against human infections, but also as preservative adjuvants in the Food and Agriculture Industry.

It is known that the use of biological agents such as EOs is an alternative to synthetic insecticides, which are not selective, are damaging to non-target organisms including humans, and lead to resistance [61]. In this sense, Cuban EOs could be useful, including the EO from *C. linearis*, which was formulated and optimized as an essential oil-loaded nanoemulsion [29]. This preparation showed a potent larvicidal effect against *Aedes aegypti* (LC_50_ = 17.8 μg/mL), without toxicity: it showed no hemolytic effect on murine erythrocytes and no cytotoxicity against human lung fibroblasts, and did not show acute oral toxicity in rats [29]. In the same way, the essential oil from *P. racemosa* was recommended for field surveillance of the use of various formulations in insect control programs of *Blattella germanica*, due to the insecticidal activity of the oil, with LD_50_ = 15.6% [39]. Finally, *Cymbopogon citratus* (DC.) Stapf EOs caused significant alterations in the post-embryonic development of *Musca domestica*, demonstrating its potential insecticidal activity [44]. 

It is worth pointing out that several Cuban EOs showed biological activities in more than one assay. For example, EO from *B. graveolens* displayed antileishmanial and antitumoral activity [27], *C. linearis* displayed antiprotozoal [28] and insecticidal activity [29], *C. sinensis* displayed antibacterial [20] and anthelmintic activity [38], *M. leucadendra* displayed antiprotozoal, antitumoral [36] and antioxidant activity [37], *M. paniculata* displayed antibacterial and antioxidant activity [45], *P. aduncum* displayed antibacterial [41] and antiprotozoal activity [40], *P. amboinicus* displayed antiprotozoal and antitumoral activity [32], and *P. auritum* displayed antileishmanial [43] and antioxidant effects [41]. These observations demonstrate that EOs have multiple biological effects or can hit a vast diversity of biological targets, probably due to their inherent chemical diversity. Certainly, the current translation of these effects towards resolving human diseases that cause a high burden in public health could be explored. In the case of antiprotozoal agents, Cuba is a country that is non-endemic to malaria, leishmaniasis and trypanosomiasis; however, imported cases have been received due to the expansion of travelers and human movement [58]. However, microbial infections {Formatting Citation} and cancer diseases [62,63] are present in the Cuban population. 

In many of these EOs, the pharmacological activity has been attributed to the main identified components (Figure 4). However, only a few pure compounds were evaluated in parallel, which is very important to confirm if the potentiality of these EOs can be attributed to the presence of the main constituents, or alternatively, is due to synergistic effects with minor components. In this sense, the biological and pharmacological properties related to the main compounds identified in EOs from Cuba are also included in Table 3. As noted, only a few studies have been conducted in the reviewed documents. For example, the monoterpene citral presented a higher mortality at all development stages of *Musca domestica* when compared to the essential oil of *C. citratus* [44]. On the other hand, 1,8 cineole showed IC_50_ values of 68.3 μg/mL (0.44 mM) and 30.3 μg/mL (0.19 mM) against *L. amazonensis* and *T. brucei*, respectively, which did not correlate with it being the main compound responsible for the antiprotozoal activity of the EO from *M. leucadendra* [36]. However, the antiproliferative activity of the oil from *P. amboinicus* could be attributed to carvacrol, which displayed IC_50_ values of 6.3 and 28.8 μg/mL against *Plasmodium falciparum* and *Leishmania amazonensis*, respectively. Against human tumor cells (MCF-7, MDA-MB-231 and 22 Rv1), carvacrol also displayed good activity, with IC_50_ values ranging from 22.1 to 36.1 μg/mL [32]. In the case of the EO from *T. lucida* [24], the observed leishmanicidal activity of the EO was also correlated with the presence of the major chemical constituent estragole, which exhibited an IC_50_ of 1.4 μg/mL (9.6 μM). 

Unfortunately, the possible mechanisms of action of bioactive EOs from Cuban plants have been only scarcely assessed. However, morphological changes in adult specimens of *Musca domestica* were described after treatment with *C. citratus* and citral [44]. The oil from *T. lucida* showed a decrease in oxygen consumption in *L. tarentolae* promastigotes and disrupted mitochondrial membrane potential in *L. amazonensis* promastigotes, while the main constituent estragole caused a decrease in the membrane potential but did not inhibit oxygen consumption in the same models [24]. Finally, the antifungal activity of EO from *Z. pseudodumosum* was explained as being due to the oxidative stress caused by the increasing of the concentration of malondialdehyde (MDA) as the mechanism of action [46]. Nevertheless, some documents highlight the antioxidant property as a feature of active EOs [7].

Often, the biological properties of EOs result from the complex interactions between the different classes of compounds. However, some studies have demonstrated that the cytotoxicity levels displayed for EOs are, in general, closely related to a few of the main components of the oils [64]. Nevertheless, the wide variation in the chemical profiles of EOs implies a great diversity in the mechanisms of action and molecular targets, which indicates that toxicity assays are needed. In this sense, most of the studies reviewed included parallel toxicity assays. In general, certain levels of safety have been demonstrated by EOs from Cuban plants. For example, for in-vitro determinations, the median cytotoxic concentrations (CC_50_) were determined and the samples could be classified as highly toxic (CC_50_ < 10 μg/mL), toxic (10 < CC_50_ < 100 μg/mL), moderately toxic (100 < CC_50_ < 1000 μg/mL) and potentially non-toxic (CC_50_ > 1000 μg/mL). Some studied EOs from Cuban plants showed a CC_50_ > 100 μg/mL, including those from: *B. graveolens* [27], *C. linearis* [29], *O. tenuiflorum* [31], *P. amboinicus* [32], *P. auritum* [43] and *T. lucida* [25]. Other EOs, using different in vivo models have demonstrated certain levels of safety, such as the EOs from *A. absinthium* [21], *C. linearis* [29], *D. ambrosioides* [18], *O. tenuiflorum* [31] and *P. carolinensis* [23]. On the other hand, in terms of the comparison between EOs and pure components, we note that the EO from *P. amboinicus* did not cause cytotoxicity at the highest tested concentration, while pure carvacrol, on the other hand, showed some cytotoxicity against mammalian cells, including MRC-5 (CC_50_ = 27.9 μg/mL) and peritoneal macrophage from BALB/c mice (CC_50_ = 32.3 μg/mL) [32]. In another case, the EO from *T. lucida* displayed CC_50_ values of 80.8 μg/mL and 156 μg/mL against peritoneal macrophages from BALB/c mice and J774 cells, respectively, while estragole itself displayed CC_50_ values of 20.6 μg/mL against peritoneal macrophages and 14.5 μg/mL against J774 cells [24].

## 3. Materials and Methods

An extensive search was carried out taking into account three settings: (i) international recognized databases—PubMed (https://pubmed.ncbi.nlm.nih.gov), Google Scholar (https://scholar.google.com), Scopus (https://www.scopus.com) and Scielo (https://www.scielo.org); (ii) Cuban electronic databases (Infomed (http://www.infomed.cu)) and relevant Cuban thematic journals (*Revista Cubana de Plantas Medicinales* (http://www.revplantasmedicinales.sld.cu) and *Revista Cubana de Farmacia* (http://www.revfarmacia.sld.cu)); and (iii) the knowledge of the research team about some articles related to essential oils from Cuba. In all cases, the combination of the keywords “essential oil” and “Cuba” was used. 

The search was conducted in April 2021 and included all reports until December 2020, without language restriction. Subsequently, a quick overview of their titles and abstracts was conducted by two different authors at same time to validate the selected articles, which were filtered to avoid duplicate papers and eliminate studies that explore plants collected outside of Cuba. Then, the full texts of the articles were downloaded and assessed to classify the type of report concerning essential oils, and they were classified within the following categories: chemical characterizations, pharmacological studies, technological approaches, or combinations of two or three of these categories. Finally, an analysis was conducted on papers that included chemical characterizations of essential oils and analyses of their biological activities, from which the data were extracted and submitted to a careful analysis. The retrieved data were used to build tables or figures to perform the bibliometric analysis and scientific review.

A bibliometric analysis was performed, which included: year, institution of first author, language and journal. However, in the compilation of characteristics of the studies and scientific results, the data extracted included: plant, geographic area of collection, part of the plant used, chemical composition and biological activity of EOs. Finally, a custom-made library was compiled to gather the compounds present in EOs and their pharmacological properties. 

## 4. Conclusions

In summary, some general considerations should be taken into account. First, there are limitations in the use of EOs that should not be ignored. For example, the chemical diversity of these products is interesting but can impact on their biological activity or specificity depending on their qualitative and quantitative compositions [65,66]. In parallel, these complex mixtures have sometimes demonstrated that several compounds could contribute synergistically to the global action of a disease. Finally, a critical point is the low yields of EOs from plant organs, which could limit their access and availability as potential drugs.

The pharmacological potentialities of these natural products could promote further studies, although it is important to highlight our appreciation that there are unknown numbers of negative results that remain unpublished. On the other hand, from this compilation, it should be taken into account that: (i) most of the reported pharmacological studies are related only to the antileishmanial, antibacterial and insecticide effects of EOs from the same team of researchers; (ii) few articles have described the advances of previously studied EOs, which limits the development of a final pharmaceutical product; (iii) the immunological and anti-inflammatory activities of EOs have not been assessed, which are common biological activities described for natural products [2,7]; and (iv) only one sample of a plant was presented in each study, which indicates that studies about the metabolomic profiles of promising plants, that include information on the different seasons or states of plants during the collection, could be of interest. In addition, although most of the results are based on in vitro studies, they are promising and suggest potential for new therapeutic opportunities. It is known that the diseases are obviously more complex than the cellular targets [4], but in vitro results will be essential for the development of EOs from Cuban plants. In addition, studies on pure compounds, synergistic and antagonistic interactions between oil components, and combinations of EOs with conventional drugs are necessary. Next, we desire to point out that in our search strategy, the general overview retrieved a higher number of articles that were related to the chemical characterization of EOs from Cuban plants, but which had not been pharmacologically studied in the same paper. A review of these data could promote further biological assessment to increase knowledge on the therapeutic potentialities of EOs from Cuban plants. 

In conclusion, we encourage further studies to explore the promising EOs as new pharmaceutical products, particularly those from *D. ambrosioides*, *C. linearis*, *M. leucadendra* and *P. aduncum*, due to their positive documented pharmacological effects and feasibility of harvest, collection and yield. Finally, this compilation could serve the Cuban scientific community as a source of inspiration to explore the nation’s diverse natural flora.

## Figures and Tables

**Table 1:** Journals that published articles related to essential oils from Cuban plants included in this review, listed in alphabetical order.

Journal	Number of Articles (%)	Impact Factor	Published	Aims/Scope
*Chemistry & Biodiversity*	3 (10)	2.408 ^a^	Wiley-VHCA AG, Zurich, Switzerland	All aspects of biologically relevant chemistry research
*Chemotherapy*	1 (3)	2.544 ^a^	Karger Publisher, Basel, Switzerland	All aspects of antimicrobial and antitumor chemotherapy
*Current Topics in Phytochemistry*	1 (3)	Not available	Research Trends, Trivandrum, India	All aspects of pure and applied plant chemistry, biochemistry, molecular biology and related interdisciplinary aspects
*Environmental Science and Pollution Research*	1 (3)	4.223 ^a^	Springer, New York, NY, USA	All areas of environmental science and related subject with emphasis on chemical compounds
*Industrial Crops and Products*	1 (3)	5.645 ^a^	Elsevier, Amsterdam, The Netherlands	Research on cultivated plants (crops) of industrial interest (non-food, non-feed)
*International Journal of Pharmacognosy and Phytochemical Research*	1 (3)	Not available	DYR Labs, Devipura, India	Research in the fields of pharmacognosy, phytochemistry, and ethnopharmacology
*ISRN Microbiology*	1 (3)	Not available	Hindawi Publishing Corporation, Cairo, Egypt	Microbiological phenomena and microbiology
*Journal of Essential Oil Research*	4 (13)	1.963 ^a^	Taylor and Francis Ltd., United Kingdom	Publication of essential oil research and analysis.
*Medicines*	1 (3)	Not available	MDPI, Basel, Switzerland	All areas of medical disciplines and sub-specialties
*Mem* *órias do Instituto Oswaldo Cruz*	1 (3)	2.070 ^b^	Fundação Oswaldo Cruz, Rio de Janeiro, Brazil	Research related to medicine (miscellaneous) or microbiology (medical)
*Molecules*	2 (7)	4.411 ^a^	MDPI, Basel, Switzerland	Provides an advanced forum for science of chemistry and all interfacing disciplines
*Natural Product Communications*	7 (23)	0.986 ^a^	SAGE Publishing, Newbury Park, CA, USA	All aspects of natural products
*Pharmacognosy Magazine*	1 (3)	1.31 ^b^	Wolters Kluwer Health, Mumbai, India	All aspects of pharmacognosy, and related fields
*Pharmacologyonline*	1 (3)	0.205 ^b^	Societa Italo-Latinoamericana di Etnomedicina, Fisciano, Italy	Pharmacology, ethnopharmacology and medicinal plants
*Phytotherapy Research*	2 (7)	5.878 ^a^	John Wiley & Sons, Hoboken, NJ, USA	Publication on medicinal plant research
*Revista Brasileira de Parasitologia Veterinária*	1 (3)	1.024 ^a^	Colégio Brasileiro De Parasitologia Veterinária, Jaboticabal, São Paulo, Brazil	Brazilian research in the areas of helminthology, protozoology, entomology and agents transmitted by arthropods, related to animal health
*Revista Cubana de Medicina Tropical*	1 (3)	0.304 ^b^	Centro Nacional de Información de Ciencias Médicas, Havana, Cuba	Publish scientific articles specialized in tropical medicine, microbiology, parasitology, epidemiology and other related specialties
*Scientia Pharmaceutica*	1 (3)	Not available	MDPI, Basel, Switzerland	All fields of pharmaceutical sciences and related disciplines

^a^ Impact factor from the journal website (accessed on 3 October 2021). ^b^ Impact factor reported by Academic Accelerator (https://academic-accelerator.com/, accessed on 3 October 2021).

**Table 2:** Main characteristics of Cuban plants investigated in publications included in this review.

Family	Plant Species	Collection Site ^a^ (Growth Conditions)	Organ Used (Yield)	Main Chemical Compounds ^f^	Ref.
Amaranthaceae	*Dysphania ambrosioides* (L.) Mosyakin & Clemants ^b^	IFAL, Havana (NR)	Fresh aerial parts (NR)	Carvacrol (62.4%) and ascaridole (22.5%)	[18]
		Caimito, Artemisa (NR)	Fresh aerial parts (NR)	α-Terpinene (20.7%), *p*-cymene (21.3%) and ascaridole (35.1%)	[19]
			Dried aerial parts (NR)	α-Terpinene (19.7%), *p*-cymene (20.2%) and ascaridole (47.1%)	
			Fermented in water aerial parts (NR)	α-Terpinene (17.0%), *p*-cymene (21.1%) and ascaridole (30.5%)	
Amaryllidaceae	*Allium sativum* L.	-	-	Di-2-propenyl trisulfide (31.9%), methyl 2-propenyl trisulfide (21.7%) and di-2-propenyl disulfide (20.7%)	[20]
Apiaceae	*Cuminum cyminum* L.	-	-	Cuminaldehyde (43.3%), cuminal (20.4%) ^f^ and β-pinene (12.8%),	[20]
	*Pimpinella anisum* L.	-	-	Anethole (80.8%)	[20]
Asteraceae	*Artemisia absinthium* L.	IFAL, Havana (NR)	Leaves (NR)	*trans*-Sabinyl acetate (36.7%)	[21]
	*Phania matricarioides* (Spreng.) Griseb.	Bauta, Artemisa (NR)	Fresh aerial parts (0.1%)	Lavandulyl acetate (40.1%) and thymyl isobutyrate (13.9%)	[22]
	*Pluchea carolinensis* (Jacq.) G. Don.	La Lisa, Havana (NR)	Fresh aerial parts (NR)	Selin-11-en-4α-ol (51.0%)	[23]
	*Tagetes lucida* Cav.	IFAL, Havana (NR)	Fresh aerial parts (NR)	Estragole (97%)	[24]
		IIIA, Havana (Cultivated)	Leaves (0.79%)	Estragole (96.8%)	[25]
Bixaceae	*Bixa orellana* L.	La Lisa, Havana (NR)	Seed (NR)	Ishwarane (18.6%) and geranylgeraniol (9.1%)	[26]
Burseraceae	*Bursera graveolens* (Kunth) Triana & Planch.	La Lisa, Havana (NR)	Fresh aerial part (NR)	Limonene (26.5%) and β-elemene (14.1%)	[27]
Euphorbiaceae	*Croton linearis* Jacq.	Reserva Ecológica Siboney-Juticí, Santiago de Cuba ^e^ (Wild)	Leaves (1.6%)	Guaiol (7.9%), guaia-3,10(14)-dien-11-ol (4.5%), selina-4(15),7(11)-diene (4.2%), and β-elemene (4.1%)	[28]
			Leaves (1.5%)	1,8-Cineole (26.7%) and sabinene (9.4%)	[29]
Fabaceae	*Tamarindus indica* L.	Santiago de Cuba ^e^ (Wild)	Leaves (NR)	Benzyl benzoate (40.9%), limonene (24.7%), and hexadecanol (11.9%)	[30]
Lamiaceae	*Ocimum tenuiflorum* L. ^c^	San Luis, Santiago de Cuba (Wild)	Leaves (0.5%)	Eugenol (22.0%), β-caryophyllene (20.8%), bicyclogermacrene (20.4%)	[31]
	*Origanum vulgare* L.,	-	-	Thymol (38.0%), *cis*-β-terpineol (16.5%), and terpinen-4-ol (10.2%)	[20]
	*Plectranthus amboinicus* (Lour.) Spreng	IFAL, Havana (Cultivated)	Fresh aerial parts (0.70–0.75%)	Carvacrol (71.0%) and *p*-cymene (9.7%)	[32]
Lauraceae	*Laurus nobilis* L.	-	-	1,8-Cineole (26.7%), eugenol (18.5%), linalool (18.5%), and sabinene (11.8%)	[20]
	*Licaria triandra* (Sw.) Kosterm.	Sierra de Meneses y Cueto, Sancti Spiritus (Wild)	Leaves (0.15%)	β-Pinene (18.2%), α-pinene (14.8%), and β-eudesmol (11.4%)	[33]
	*Nectandra hihua* (Ruiz & Pav.) Rohwer ^d^	Sierra de Meneses y Cueto, Sancti Spiritus (Wild)	Leaves (0.39%)	Caryophyllene oxide (16.0%) and β-caryophyllene (9.9%)	[34]
Myrtaceae	*Callistemon speciosus* (Sims) Sweet	Candelaria, Pinar del Río (Wild)	Leaves (0.93%)	1,8-Cineole (57.0%) and α-terpineol (20.4%)	[35]
	*Melaleuca leucadendra* L.	NBG, Havana (Cultivated)	Fresh aerial parts (0.8%)	1,8-Cineole (61.0%) and α-terpineol (15.6%),	[36]
		Ciénaga de Zapata, Matanzas (Wild)	Leaves (0.7%)	1,8-Cineole (43.0%) and viridiflorol (24.2%)	[37]
			Fruit (0.4%)	Viridiflorol (47.6%)	
	*Melaleuca quinquenervia* (Cav.) S.T. Blake	-	-	Longifolene (32.9%) and 1,8-cineole (25.4%)	[38]
	*Pimenta racemosa* (Mill.) J.W. Moore	Pinar del Río (Wild)	Leaves (NR)	Terpinen-4-ol (20.7%), 1,8-cineole (20.4%), eugenol (10.7%), and α-terpineol (10.0%)	[39]
	*Syzygium aromaticum* (L.) Merr. & L.M. Perry	-	-	Eugenol (67.0%) and eugenyl acetate (18.1%)	[20]
Piperaceae	*Piper aduncum* L.	IFAL, Havana (NR)	Fresh aerial parts (NR)	Piperitone (23.7%), camphor (17.1%), and viridiflorol (14.5%)	[40]
		Topes de Collantes, Sancti Spiritus (Wild)	Leaves (1.3%)	Piperitone (34.0%) and camphor (17.1%)	[41]
	*Piper aduncum* subsp. *ossanum* Trel.	Bauta, Artemisa (Wild)	Leaves (0.48%)	Piperitone (20.1%), camphor (13.9%) and viridiflorol (13.0%)	[42]
		Caimito, Artemisa (Wild)	Leaves (0.37%)	Piperitone (%), camphor (19.0%) and viridiflorol (18.8%)	
	*Piper auritum* Kunth	IFAL, Havana (NR)	Fresh aerial part (NR)	Safrole (87%)	[43]
		Topes de Collantes, Sancti Spiritus (Wild)	Leaves (2.5%)	Safrole (71.8%)	[41]
	*Piper umbellatum* L.	Topes de Collantes, Sancti Spiritus (Wild)	Leaves (2.0%)	Safrole (26.4%) and camphor (9.6%)	[41]
Poaceae	*Cymbopogon citratus* (DC.) Stapf	Moa, Holguín (Wild)	Leaves (NR)	Geranial (51.1%) and neral (35.2%)	[44]
Rutaceae	*Citrus sinensis* (L.) Osbeck	-	-	Limonene (82.7%)	[20]
		-	-	Limonene (96.0%)	[38]
	*Murraya paniculata* (L.) Jack	Topes de Collantes, Sancti Spiritus (Cultivated)	Leaves (0.2%)	β-Caryophyllene (29.8%)	[45]
	*Zanthoxylum pseudodumosum* Beurton	Camajuaní, Santa Clara (Wild)	Leaves (0.43%)	β-Caryophyllene (32.0%) and germacrene D (14.9%)	[46]
Verbenaceae	*Lantana camara* L.	Caimito, Artemisa (NR)	Fresh aerial part (0.3%)	(*E*)-Nerolidol (16.6%) and (*E*)-β-farnesene (11.3%)	[47]
Zingiberaceae	*Alpinia zerumbet* (Pers.) B.L. Burtt & R.M. Smith	NBG, Havana (Cultivated)	Leaves (0.25%)	Terpinen-4-ol (19.0%) and caryophyllene oxide (18.2%)	[48]
			Flowers (0.20%)	Terpinen-4-ol (14.1%) and viridiflorol (32.2%)	

^a^ Place or municipality, Province. ^b^ *Chenopodium ambrosioides* L. ^c^ *Ocimum sanctum* L. ^d^ *Nectandra antillana* Meisn. ^e^ Names of Municipality and Province are the same. ^f^ Chemical structures of the most common components are represented in Figure 4. ^f^ Cuminaldehyde and cuminal are synonymous; this report is in error. -: No data were included; NR: Not Reported. IFAL: Institute of Pharmacy and Food, La Lisa. IIIA: Food Industry Research Institute, La Lisa. NBG: National Botanic Garden, Arroyo Naranjo.

**Table 3:** Pharmacological studies of essential oils and main compounds from Cuban plants included in this review.

Plant Species	Pharmacological Property	Model	Species/Activity (Main Compound Assayed)	Ref.
*A. absinthium*	Antileishmanial activity	In vitro and In vivo	- *Leishmania amazonensis*	[21]
*A. sativum*	Antibacterial activity	In vitro	- *Enterobacter agglomerans* - *Streptomyces* sp.	[20]
*A. zerumbet*	Antiplasmodial activity	In vitro	- *Plasmodium berghei*	[48]
*B. graveolens*	Antileishmanial activity and antitumoral effect	In vitro	- *Leishmania amazonensis* - MCF-7 breast tumor cells	[27]
*B. orellana*	Antileishmanial activity	In vitro and in vivo	- *Leishmania amazonensis*	[26]
*C. citratus*	Antifungal and insecticide effects	In vivo	- *Musca domestica* (citral)	[44]
*C. cyminum*	No relevant activity	In vitro	Antimicrobial assessment	[20]
*C. linearis*	Antileishmanial, antitrypanosomal and larvicidal effect	In vitro	- *Leishmania amazonensis* - *Trypanosoma cruzi*	[28]
		In vivo	- Aedes aegypti	[29]
*C. sinensis*	Antibacterial activity	In vitro	- *Streptomyces* sp.	[20]
	Anthelmintic effects	In vitro and in vivo	- *Haemonchus contortus*	[38]
*C. speciosus*	Antibacterial activity	In vitro	- *Bacillus cereus* - *Bacillus subtilis* - *Escherichia coli* - *Listeria monocytogenes* - *Staphylococcus aureus*	[35]
*D. ambrosioides*	Antileishmanial activity	In vitro and in vivo	- *Leishmania amazonensis*	[18]
		In vitro	- *Leishmania amazonensis*	[19]
*L. camara*	Antibacterial activity	In vitro	- *Staphylococcus aureus*	[47]
*L. nobilis*	No relevant activity	In vitro	Antimicrobial assessment	[20]
*L. triandra*	Antibacterial activity	In vitro	- *Bacillus cereus* - *Bacillus subtilis* - *Escherichia coli* - *Listeria monocytogenes* - *Staphylococcus aureus*	[33]
*N. hihua*	Antibacterial activity	In vitro	- *Bacillus cereus* - *Escherichia coli* - *Listeria monocytogenes* - *Staphylococcus aureus*	[34]
*M. leucadendra*	Antileishmanial, antitrypanosomal, antitumoral and antioxidant activity	In vitro	- *Leishmania amazonensis* (1,8-cineole) - *Trypanosoma brucei* (1,8-cineole) - Malignant cell lines 22 Rv1, MCF-7, EFO-21, MCF-7/Rap and MCF-7/4OHTAMO	[36]
		In vivo	- *Leishmania amazonensis*	
		In vitro	Antioxidant activity	[37]
*M. paniculata*	Antibacterial and antioxidant effect	In vitro	- Antioxidant activity - *Klebsiella pneumoniae* - *Bacillus subtilis*	[45]
*M. quinquenervia*	Anthelmintic effects	In vitro and in vivo	- *Haemonchus contortus*	[38]
*O. tenuiflorum*	No relevant activity	In vitro and in vivo	- Toxicity assay that demonstrated safety of essential oil	[31]
*O. vulgare*	Antibacterial activity	In vitro	- *Enterobacter agglomerans* - *Streptomyces* sp.	[20]
*P. aduncum*	Antiprotozoal and antibacterial activity		- Lower antioxidant activity	[41]
		In vitro	- *Plasmodium falciparum* - *Trypanosoma brucei* - *Trypanosoma cruzi* - *Leishmania amazonensis* - *Leishmania donovani* - *Leishmania infantum* - *Staphylococcus aureus*	[40]
*P. amboinicus*	Antiprotozoal and antitumoral activity	In vitro	- *Plasmodium falciparum* (carvacrol) - *Trypanosoma brucei* - *Leishmania amazonensis* (carvacrol) - Malignant cell lines MCF-7, MDA-MB-231 and 22 Rv1 (carvacrol)	[32]
*P. anisum*	Antifungal against	In vitro	- *Aspergillus niger* - *Aspergillus clavatus* - *Penicillium* sp. - *Fusarium* sp.	[20]
*P. auritum*	Antileishmanial and antioxidant activity	In vitro	- *Leishmania major* - *Leishmania mexicana* - *Leishmania braziliensis* - *Leishmania donovani*	[43]
		In vitro	- Antioxidant activity	[41]
*P. carolinensis*	Antileishmanial activity	In vitro and in vivo	- *Leishmania amazonensis*	[23]
*P. matricarioides*	Antiprotozoal activity	In vitro	- *Plasmodium falciparum* - *Trypanosoma brucei* - *Trypanosoma cruzi* - *Leishmania amazonensis* - *Leishmania infantum*	[22]
*P. ossanum*	Antiprotozoal activity	In vitro	- *Plasmodium falciparum* - *Trypanosoma brucei* - *Trypanosoma cruzi* - *Leishmania amazonensis* - *Leishmania infantum*	[54]
*P. racemosa*	Insecticidal effect	In vivo	- *Blattella germanica*	[39]
*P. umbellatum*	No relevant effect	In vitro	- Lower antioxidant activity	[41]
*S. aromaticum*	Antibacterial activity	In vitro	- *Enterobacter agglomerans* - *Streptomyces* sp.	[20]
*T. indica*	Antibacterial and antifungal activity	In vitro	- *Bacillus subtilis* - *Candida albicans* - *Enterobacter faecalis* - *Escherichia coli* - *Staphylococcus aureus* - *Salmonella typhimurium*	[30]
*T. lucida*	Antileishmanial activity	In vitro	- *Leishmania tarentolae* (estragole) - *Leishmania amazonensis* (estragole)	[24]
	Antiplasmodial and antibacterial activity	In vitro	- *Plasmodium berghei* - *Escherichia coli*	[25]
*Z. pseudodumosum*	Antifungal activity	In vitro	- *Alternaria solani*	[46]

## Data Availability

All data are available in the article.

## Supplementary Materials

The following are available online at https://www.mdpi.com/article/10.3390/plants10112515/s1, Figure S1: Flowchart of the screening process to select articles included in this review.
