# Supplementary figures and images for: Bioactive Essential Oils from Cuban Plants: An Inspiration to Drug Development
Source: Plants (Basel). 2021 Nov 19;10(11):2515. doi: 10.3390/plants10112515 (PMC8620706; doi:10.3390/plants10112515)

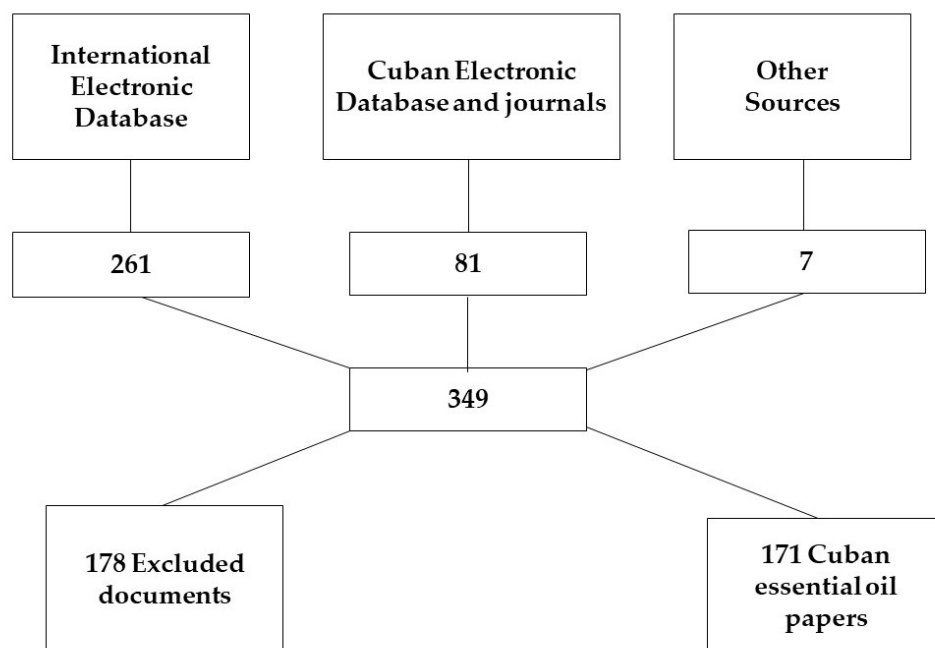

**Figure S1.** Flowchart of the screening process to select articles included in this review.

Supplement: Supplementary file 1 [file plants-10-02515-s001.zip › plants-1428128-supplementary.pdf]
